# Paleopathology Meets Public Health: Deep-Time Syndemics and the Ecology of Emerging Infections

**DOI:** 10.3390/pathogens15050543

**Published:** 2026-05-18

**Authors:** Hisham F. Bahmad, Ghassan Ghssein, Marwan Bahmad, Tarec K. Elajami, Irman Forghani, Claudio Tuda, Roberto Ruiz-Cordero

**Affiliations:** 1 Department of Pathology and Laboratory Medicine, University of Miami Miller School of Medicine, Miami, FL 33136, USA; rxr1314@med.miami.edu; 2 High Council for Scientific Research & Publication (HCSRP), Islamic University of Lebanon, Khalde P.O. Box 30014, Lebanon; ghassan.ghssein@iul.edu.lb; 3 Division of Vascular Surgery, Department of Surgery, American University of Beirut Medical Center, Beirut 1107, Lebanon; mb212@aub.edu.lb; 4 Semyon and Janna Friedman Advanced Research Institute, Mount Sinai Medical Center, Miami Beach, FL 33140, USA; tarec.elajami@msmc.com; 5 Division of Cardiology, Mount Sinai Medical Center, Miami Beach, FL 33140, USA; 6 New York Medical College School of Medicine, Valhalla, NY 10595, USA; 7 Department of Clinical Genetics, Mount Sinai Medical Center of Florida, Miami Beach, FL 33140, USA; irman.forghani@msmc.com; 8 Department of Internal Medicine, Mount Sinai Medical Center, Miami Beach, FL 33140, USA; claudio.tuda@msmc.com

**Keywords:** paleopathology, public health, one health, pandemics, antimicrobial resistance

## Abstract

Why do pandemics keep emerging despite decades of surveillance and response? Paleopathology, the study of disease traces in ancient remains, has been revolutionized by ancient DNA (aDNA) analysis and next-generation sequencing (NGS). Reconstructing pathogen genomes from archaeological material enables the identification of extinct lineages, the refinement of disease chronologies, and the characterization of long-term host-pathogen co-evolution. This provides context for public health challenges, including the emergence of pandemics and antimicrobial resistance (AMR). Infectious diseases are increasingly understood as complex phenomena arising from biological, ecological, and sociopolitical forces. Integrating paleopathology, aDNA, and paleomicrobiology supports a deep-time syndemic framework, revealing how recurring biosocial drivers have structured infectious disease risk throughout history. Ancient resistome studies demonstrate that AMR predates modern antibiotic use, reframing resistance as an intrinsic ecological feature rather than solely a modern phenomenon. Coronavirus disease 2019 (COVID-19) reaffirmed how infection intersects with chronic disease, health system fragility, and social inequities. This review highlights how integrating evolutionary perspectives into One Health shifts surveillance from a reactive approach to upstream risk mitigation and spillover prevention.

## 1. Introduction

The accelerating pace of emerging and re-emerging infectious diseases in the twenty-first century has exposed the limitations of outbreak models that frame pandemics as isolated biological events [1]. Contemporary evidence shows that pathogen emergence is driven by interacting biological, ecological, and sociopolitical forces, including climate change, land-use transformation, urbanization, armed conflict, population displacement, and structural health inequities [2,3,4,5,6]. In response, global health frameworks and initiatives have increasingly adopted integrative approaches, such as One Health and syndemic theory, to address the complexity of disease emergence at the human–animal–environment interface, largely in recognition that traditional siloed approaches failed during recent pandemics [6,7].

Syndemic theory emphasizes that diseases cluster within populations under specific social and environmental conditions, and that these diseases interact biologically and behaviorally, amplifying morbidity and mortality beyond the effects of individual diseases [8,9]. The One Health framework similarly argues that pandemic prevention requires addressing upstream ecological and anthropogenic drivers of spillover, rather than relying exclusively on downstream biomedical responses [10,11,12]. Despite their conceptual alignment, both approaches remain largely forward-looking and are insufficiently informed by long-term historical evidence.

Paleopathology and ancient pathogen genomics offer the empirical foundation needed to bridge this gap. By providing a deep-time perspective, spanning thousands to millions of years of human–pathogen interaction, as the term is used in geology and evolutionary biology, these disciplines reveal that the biosocial configurations driving disease emergence today are not unprecedented but represent recurring patterns with deep evolutionary roots [13,14]. A syndemic, defined as the synergistic interaction of two or more diseases or health conditions within a population driven by social and environmental forces, becomes far more tractable when situated within this long temporal arc. This review proposes a deep-time syndemic framework that uniquely integrates empirical evidence from paleopathology and archaeogenomics with syndemic theory and One Health principles, adding a temporal depth dimension absent from existing ecosocial and One Health models, to reconceptualize pandemic preparedness as rooted in evolutionarily recurring biosocial configurations.

The One Health model has evolved from narrow veterinary–medical cooperation to a comprehensive transdisciplinary framework that encompasses human, animal, plant, and environmental health within social–ecological systems [15]. This evolution accelerated dramatically following COVID-19, with the 2010 Tripartite (WHO, FAO, WOAH) expanding to the Quadripartite in March 2022 by adding the United Nations Environment Programme (UNEP) [6,15]. The One Health High-Level Expert Panel (OHHLEP), established in 2020, provided a unified definition emphasizing the “integrative and transdisciplinary approach needed to coordinate actors from a wide range of disciplines beyond human and domestic animal health” [16]. In view of that, the syndemic theory has been integrated into One Health frameworks to capture how multiple afflictions interact synergistically at the human–animal interface [17,18]. This approach recognizes that disease emergence involves not just pathogen spillover but also the convergence of biological, social, and environmental factors, acknowledging that “the entry points for One Health issues frequently begin with human behaviors, our interactions with the environment, and wider ecosystem stability” [18].

Ancient DNA (aDNA) and paleomicrobiology now enable direct observation of how subsistence transitions, animal domestication, population aggregation, and ecological disruption reshaped infectious disease landscapes from the Late Pleistocene through the Anthropocene. This molecular archive has yielded complete genomes for major historical pathogens, including *Yersinia pestis* (*Y. pestis*), *Variola virus*, *Vibrio cholerae* (*V. cholerae*), *Mycobacterium tuberculosis* (*M. tuberculosis*), *Mycobacterium leprae* (*M. leprae*), and *Treponema pallidum* (*T. pallidum*) [14,19]. These data have accomplished what historical texts cannot: the confident identification of causative agents from past pandemics, the discovery of extinct microbial lineages, precise chronological dating of pathogen emergence, and the characterization of host–pathogen co-evolution relevant to contemporary public health [14,20,21].

The Neolithic transition, or revolution, offers the most compelling evidence linking human behavioral change to pathogen emergence [22,23,24,25]. Large-scale archaeogenomic screening of 1313 ancient humans spanning 37,000 years of Eurasian history revealed that zoonotic pathogens emerged around 6500 years ago and peaked about 5000 years ago, coinciding with the onset of widespread livestock domestication [24]. This timing provides direct molecular evidence that the shift to agriculture and animal husbandry increased the infectious disease burden, not merely through population growth but through novel human–animal interfaces that enabled spillover [22,26]. For instance, human-adapted *Salmonella enterica Paratyphi C* evolved over 5000 years from non-host-specific strains found among transitional foragers and early agropastoralists, demonstrating convergent pseudogenization accompanying host adaptation during the Neolithization process [22]. Another example is the Bronze Age *Y. pestis* genome recovered from a 3000 BCE domesticated sheep (belonging to a lineage previously identified only in ancient humans) that directly links prehistoric livestock to human infection risk, showing how pathogens spread from unidentified reservoirs to domesticates and subsequently to humans [26,27].

These paleogenomic findings validate syndemic and One Health predictions by demonstrating that pathogen emergence results from convergent biological, ecological, and social forces operating over millennia, rather than from linear causation. The archaeological records from Neolithic Çatalhöyük (7100–5950 cal BCE), a massive archaeological site in south-central Anatolia (Turkey), illustrate this complexity [28,29]. Bioarchaeological analysis revealed elevated disease exposure, increased fertility driving population density, heightened labor demands from crop production, and greater mobility for animal herding, which is a biosocial configuration remarkably similar to contemporary urbanization, intensive agriculture, and pastoral migration patterns [28].

Paleopathology thus provides what forward-looking frameworks lack: empirically grounded observations of recurring “outbreak modules” characterized by ecological disruption, population aggregation or displacement, sanitation challenges, and health system stress. Ancient pathogen genomics reveals that these configurations are not historically bounded but represent evolutionarily stable conditions that facilitated pathogen success across vastly different time periods and geographic contexts [13,19,30]. The field demonstrates that anthropogenic environmental modification (not just domestication per se) may be the broader evolutionary force shaping human–pathogen relationships, a finding with direct relevance to contemporary climate change and land-use transformation [25].

The relevance to modern pandemic preparedness is substantial. Molecular typing of ancient pathogens enables the reconstruction of past epidemic transmission routes and evolutionary trajectories, informing current models of emerging infections and contributing to the design of surveillance systems. Historical bacterial genomes from collections spanning key historical events provide phylodynamic insights that are particularly valuable for understanding the evolution of antimicrobial resistance (AMR). Studies emphasized that ancient pathogen genomics is “poised to benefit current public health challenges” by revealing mechanisms underlying pathogen success, host susceptibility, and the genetic, cultural, and ecological factors that affect disease dynamics [13,21,30].

This review has four specific objectives: (1) to demonstrate how paleopathological and archaeogenomic evidence reveals recurring biosocial configurations (termed “outbreak modules” and defined as recurring configurations characterized by ecological disruption, population aggregation or displacement, sanitation collapse, and health system stress) that have driven infectious disease emergence across millennia; (2) to reframe AMR as a deep-time ecological phenomenon rather than solely a modern consequence of antibiotic misuse; (3) to analyze how conflict, displacement, and climate change function as syndemic amplifiers with deep historical precedents; and (4) to propose an integrative deep-time syndemic framework linking paleopathology with One Health principles for improved pandemic preparedness.

**Figure 1:**
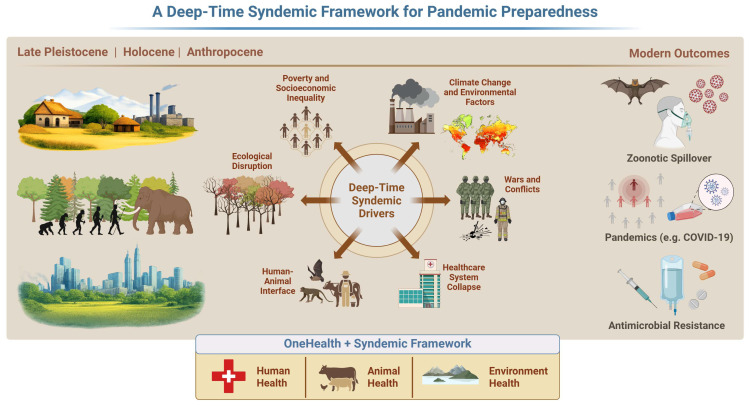
A deep-time syndemic framework for pandemic preparedness. Schematic representation illustrating how recurring biosocial drivers of infectious disease emergence operate across deep time, from the Late Pleistocene and Holocene to the Anthropocene. Early transitions in human subsistence, mobility, settlement patterns, and human–animal interactions established persistent ecological conditions that facilitated pathogen spillover and long-term host–pathogen co-evolution. Central “deep-time syndemic drivers” include ecological disruption, intensified human–animal interfaces, poverty and socioeconomic inequality, climate change and environmental stressors, armed conflict, and healthcare system collapse, which interact synergistically rather than independently. These drivers converge to produce outcomes such as zoonotic spillover, pandemics (e.g., COVID-19), and amplification of antimicrobial resistance, disproportionately affecting structurally vulnerable populations. The lower panel highlights the integrative One Health plus syndemic framework, emphasizing the interconnected roles of human, animal, and environmental health in surveillance, prevention, and pandemic preparedness. Created in BioRender.

Building on this evidence, this review provides a basis for a deep time syndemic framework that integrates paleopathological and archaeogenomic data with syndemic theory and One Health principles (Figure 1). We argue that many historical outbreaks conventionally framed as discrete “pathogen events” were, in fact, products of recurring biosocial configurations (here termed outbreak modules) characterized by ecological disruption, population aggregation or displacement, sanitation collapse, and health system failure. These same configurations are re-emerging today under climate change, armed conflict, mass migration, and ecological degradation, accelerating zoonotic spillover and amplifying health inequities. By situating modern pandemic risk within long-term evolutionary and ecological contexts, paleopathology provides empirically grounded insights essential for surveillance, early warning systems, and primary prevention strategies.

This is a narrative and integrative review, not a systematic review, which we consider appropriate given the trans-disciplinary scope of the manuscript. Literature was identified through comprehensive searches of PubMed, Web of Science, Scopus, and Google Scholar using key terms including paleopathology, ancient DNA, paleomicrobiology, syndemic theory, One Health, antimicrobial resistance, archaeogenomics, and related terms. The review does not claim to be exhaustive but aims to synthesize the most relevant evidence across disciplines to support the proposed deep-time syndemic framework.

## 2. From Paleopathology to Genomic Paleoepidemiology

Classical paleopathology traditionally relied on macroscopic and histological examinations of skeletal remains to infer the presence of disease, focusing on characteristic bony lesions associated with chronic infections such as tuberculosis (TB), treponematoses, and leprosy. While foundational, this approach is inherently limited since many infections leave no skeletal trace. For instance, TB produces bone lesions in only 3–5% of cases, and numerous acute infections cause death before skeletal changes can develop [31,32,33]. Even when lesions are present, morphological overlap between diseases complicates definitive diagnosis.

The integration of microbiological techniques into archaeological research marked a critical transition. In addition, molecular techniques have substantially expanded diagnostic capabilities beyond what skeletal lesions alone can reveal. Ancient DNA analysis has identified TB in individuals with nonspecific or absent skeletal pathology, detected co-infections with multiple pathogens, and revealed diseases like malaria that were previously thought absent from certain populations [32,34]. Early molecular studies using polymerase chain reaction (PCR) and immunodetection enabled the identification of pathogen DNA and antigens from dental pulp, bone, coprolites, and mummified tissues, establishing proof-of-concept that infectious agents could be detected directly in ancient human remains [35]. However, these approaches s have always been constrained by contamination risk, limited genomic resolution, and small sample sizes.

High-throughput sequencing and stringent authentication criteria have fundamentally transformed paleopathology from a morphology-based discipline into genomic paleoepidemiology by enabling the recovery and validation of complete ancient pathogen genomes from archaeological remains, even when skeletal lesions are absent or nonspecific [13,14,20,21,36]. Metagenomic approaches have been pivotal in reconstructing near-complete genomes of historically significant pathogens [37]. Next-generation sequencing (NGS) technologies allow preferential capture and amplification of discrete pathogen genomes from backgrounds dominated by host and environmental DNA [20]. This has yielded complete or near-complete ancient genome sequences for many pathogens, including but not limited to *Y. pestis*, *Variola virus*, *V. cholerae*, *M. tuberculosis*, *M. leprae*, *T. pallidum*, and hepatitis B virus [20,21,38].

The significance extends beyond pathogen identification to evolutionary reconstruction. Ancient pathogen genomics provides a molecular fossil record that enables identification of now-extinct lineages, refinement of pathogen emergence chronology, and characterization of long-term evolutionary trajectories [14,21]. For example, metagenomic analysis of Egyptian mummies successfully reconstructed a 2200-year-old *M. leprae* genome and a 2000-year-old hepatitis B virus genome [38].

Nevertheless, authentication criteria are essential for reliability. The field has developed specialized methods to authenticate aDNA and quantify contamination, challenges that previously plagued paleopathology [14,36]. These stringent standards, combined with the analytical power of billions of sequence reads, enable the confident identification of causative agents from past pandemics and the accurate reconstruction of pathogen evolution over large timescales [13,14]. This transformation enabled tracking pathogen evolution “in action” across time, revealing patterns of emergence, geographic spread, and host–pathogen interactions that inform current public health challenges [13,39].

Several important methodological limitations must be acknowledged when interpreting paleogenomic findings. First, the osteological paradox highlights that skeletal samples represent a biased subset of past populations: individuals whose skeletons appear healthy may have died too acutely for lesions to develop, while those with visible pathology may represent survivors of chronic infection rather than the most severely affected. Second, preservation biases strongly favor cold and dry environments, meaning that tropical and equatorial regions, which are critical for understanding modern disease emergence, remain substantially underrepresented in the ancient DNA record. Third, contamination with modern DNA remains a persistent challenge, despite increasingly stringent authentication protocols. Fourth, the absence of soft tissue and immune response data limits inference about host susceptibility, disease severity, and immune interactions in past populations. Finally, geographic sampling remains heavily skewed toward Eurasia, constraining generalizability to other regions. These limitations should be kept in mind when evaluating the extrapolation of paleogenomic findings to contemporary epidemiological contexts throughout this review.

Large-scale archaeogenetic screening has fundamentally validated and refined the epidemiological transition hypothesis by providing direct molecular evidence linking subsistence changes to pathogen landscapes. A landmark 2025 study analyzing shotgun sequencing data from 1313 ancient individuals spanning approximately 37,000 years across Eurasia identified 5486 individual pathogen hits against 492 species, with 3384 involving known human pathogens, many never previously identified in ancient remains [24].

Zoonotic pathogens were detected only after animal domestication began, appearing around 6500 years ago and peaking approximately 5000 years ago, coinciding precisely with widespread livestock domestication [24]. This provided direct molecular evidence supporting the first epidemiological transition hypothesis that the shift from hunting-gathering to agriculture fundamentally altered human pathogen exposure [40]. For example, aDNA from an 8000-year-old sheep infected with *Brucella melitensis* (*B. melitensis*) demonstrated that zoonotic pathogens had evolved and were circulating in Neolithic livestock, with speciation from cattle-infecting strains occurring around 9800 years before present [41].

Long-term skeletal analyses revealed host–pathogen coevolution rather than eradication. Meta-analyses of tens of thousands of skeletons spanning more than 200 human generations (approximately 69,379 individuals) showed statistically significant declines in skeletal manifestations of TB, leprosy, and treponematoses over time (*p* < 0.001) [33]. This pattern reflects reciprocal adaptation (pathogens persisting with reduced virulence while hosts develop tolerance or partial immunity) rather than disease elimination [33,42]. In fact, co-infections of *M. tuberculosis* and *M. leprae* detected in archaeological samples suggest that immunological interactions between these pathogens may have contributed to leprosy’s historical decline in Europe [34,43].

Interestingly, integration with archaeological context also enabled the identification of biosocial disease drivers. When pathogen diversity is examined alongside mobility patterns, settlement disruption, warfare, sanitation, and animal exploitation, recurring configurations associated with heightened disease burden emerge [13,19,44]. The spread of zoonotic pathogens increased substantially during pastoralist migrations from the Eurasian Steppe, demonstrating how human mobility amplified pathogen transmission [24]. This integrative capacity positions genomic paleoepidemiology as critical for understanding (and potentially anticipating) modern patterns of infectious disease emergence by revealing how human behavioral and environmental changes have repeatedly shaped pathogen evolution over millennia.

In summary, the transformation of paleopathology into genomic paleoepidemiology has provided unprecedented empirical evidence for understanding the deep evolutionary origins of infectious disease emergence. These findings establish the evidential foundation for the deep-time syndemic framework by demonstrating that pathogen emergence has been consistently shaped by human behavioral and environmental changes over millennia. The following section examines how the first epidemiological transition, the Neolithic Revolution, serves as a concrete template for understanding modern spillover risk through these same biosocial mechanisms.

## 3. The First Epidemiological Transition as a Template for Modern Spillover Risk

The epidemiological transition is a theoretical framework describing the shift in disease patterns and mortality causes that accompanies demographic and socioeconomic development, originally proposed by Abdel Omran in 1971 [45]. The concept extends the demographic transition (declining fertility and mortality with population aging) to encompass the characteristic shifts in leading causes of death and morbidity over time, providing a foundational framework for understanding how large-scale changes in human ecology and social organization reshape disease patterns. The first epidemiological transition is directly associated with the Neolithic Revolution, which occurred approximately 10,000 years ago, when human populations shifted from mobile foraging to sedentary agriculture and the domestication of livestock [40,46]. This transition provided a validated deep-time template for understanding modern spillover risk by demonstrating that zoonotic disease emergence follows reproducible patterns tied to human–animal–environment interactions, population aggregation, and mobility, patterns that remain highly relevant in contemporary contexts [24,40,46].

The demographic consequences were profound. The Neolithic Demographic Transition resulted in an estimated increase of two births per woman, evidenced by an abrupt rise in the proportion of juvenile skeletons in cemetery data from archaeological sequences worldwide [28,47,48]. This population expansion occurred despite deteriorating health markers, including increased infectious disease burden, nutritional deficiencies, and reduced adult stature.

Ancient DNA evidence confirmed that the Neolithic transition fundamentally altered pathogen landscapes, providing molecular validation that sustained animal contact, population density, and mobility are prerequisites for large-scale zoonotic transmission. Crucially, the transition was not driven by a single factor, but by a constellation of interacting changes. Domesticated animals served as new reservoirs and amplification hosts; permanent settlements increased population density and contact rates; waste accumulation and water contamination undermined sanitation; and emerging social stratification created differential exposure and vulnerability. Together, these factors formed an early biosocial configuration that repeatedly generated infectious disease risk [28,40,49]. Importantly, ancient pathogen genomics indicates that pathogen emergence during this period was not uniform but episodic, intensifying during phases of ecological stress, migration, or sociopolitical instability, closely mirroring modern outbreak dynamics.

From a contemporary perspective, the first epidemiological transition serves as a deep-time analog for modern spillover risk. Industrial agriculture, wildlife trade, deforestation, extractive industries, and peri-urban expansion have dramatically intensified human–animal contact at scales far exceeding those of early farming societies. Similarly, modern mobility (through global travel, labor migration, and forced displacement) has compressed spatial and temporal barriers to transmission. The archaeological record demonstrates that when societies scaled animal contact and mobility without commensurate investments in sanitation, health infrastructure, and governance, pathogen landscapes shifted measurably and persistently [50]. These dynamics are directly observable today in regions undergoing rapid land-use change and socioeconomic transformation.

The One Health framework emphasizes that spillover prevention must focus on upstream drivers at the human–animal–environment interface rather than on reactive outbreak response. Deep-time evidence strengthens this argument by showing that spillover is not an anomalous event but a predictable outcome of specific ecological and social configurations. Ancient pathogen maps serve as a natural experiment spanning millennia, revealing that similar combinations of animal contact, environmental disruption, and population movement repeatedly produced zoonotic risk long before modern globalization.

Importantly, the first epidemiological transition also illustrates that pathogen emergence is path-dependent [51]. Once established, zoonotic pathogens can persist for a long period of time, adapting alongside human hosts and reshaping disease ecologies long after the original ecological trigger has passed [4]. This insight challenges linear narratives of emergence and control, signifying the need for prevention strategies that address structural drivers rather than individual pathogens. In this context, modern spillover events, whether involving coronaviruses, influenza viruses, or hemorrhagic fevers, should be understood not as unprecedented crises but as contemporary expressions of deep, recurring biosocial processes that began with the Neolithic transition and continue today [52,53].

By reframing the first epidemiological transition as a template rather than a historical curiosity, paleopathology and archaeogenetics provide a powerful lens for anticipating modern spillover risk. They demonstrate that zoonotic emergence follows reproducible patterns tied to human behavior, environmental change, and social organization, patterns that remain highly relevant in the Anthropocene and that demand integrative, prevention-focused responses.

The first epidemiological transition thus demonstrates that zoonotic disease emergence follows reproducible patterns tied to human–animal–environment interactions. These same biosocial forces shaped not only pathogen emergence but also the evolution of AMR, a phenomenon now understood to have ancient ecological origins that long predate modern antibiotic use, as examined in the following section.

## 4. The Ancient Resistome: Reframing Antimicrobial Resistance as a Deep-Time Ecological Phenomenon

### 4.1. Antimicrobial Resistance Beyond Modernity: Evidence from Deep Time

AMR is widely interpreted as a distinctly modern consequence of antibiotic misuse and overuse; however, paleomicrobiological and environmental genomic evidence fundamentally challenges this narrative [54,55]. Ancient DNA and metagenomic analyses demonstrate that resistance determinants predate clinical antibiotic use by millennia, reframing AMR as an ancient, evolutionarily conserved ecological phenomenon that has been dramatically amplified (but not created) by contemporary selection pressures [31,56].

### 4.2. Environmental Origins of Resistance: Permafrost, Caves, Soils, and Sediments

Resistance genes have been recovered from a wide range of ancient and minimally disturbed environments, including permafrost, deep caves, isolated soils, and archaeological human remains [57,58]. Functional resistance mechanisms (target modification, efflux pumps, enzymatic degradation, and reduced permeability) mirror those observed in modern clinical pathogens, indicating that resistance emerged as a natural outcome of microbial competition and chemical warfare long before the Anthropocene [59,60,61]. Antibiotic-producing organisms and their competitors have coexisted for millions of years, generating a vast environmental resistome that serves as a reservoir of adaptive potential [62]. Importantly, advances in diagnostic techniques for infectious diseases, including multiplex PCR panels and whole-genome and whole-exome sequencing, have shortened the time to identify the causative agent and, in bacterial infections, enabled the detection of specific antimicrobial resistance genes (ARGs). These capabilities can play an important role in tracking AMR in both community- and hospital-associated cases and can help guide the selection of suitable treatment, particularly in empirical settings [63,64].

Ancient human-associated microbiomes further demonstrate that resistance genes circulated within human populations prior to modern medicine [30]. Paleofecal material, dental calculus, and mummified tissues contain resistance determinants analogous to contemporary β-lactamases, tetracycline resistance genes, and multidrug efflux systems, indicating that early human-microbe ecosystems already harbored functionally diverse resistomes [65]. Phylogenetic analyses indicate β-lactamases emerged 1–2 billion years ago, and permafrost samples from the Yukon revealed bacteria with resistance mutations 30,000 years before penicillin’s discovery [61]. These findings directly contradict linear models in which resistance is viewed as a post-antibiotic aberration, instead positioning AMR as a latent ecological trait that becomes clinically relevant under specific social and environmental conditions.

Isolated cave ecosystems demonstrate ancient resistance independent of human antibiotic exposure. Lechuguilla Cave, New Mexico, isolated for over 4 million years, harbored culturable bacteria resistant to 14 different commercially available antibiotics, including daptomycin, an antibiotic of last resort [57]. Resistance mechanisms included enzyme-mediated glycosylation and kinase-mediated phosphorylation of macrolide antibiotics, and genome sequencing revealed macrolide kinase genes related to those found in modern drug-resistant pathogens [57]. This further confirms that AMR is “natural, ancient, and hard-wired in the microbial pangenome” [57].

Importantly, recent studies highlight that the value of ancient resistome research extends beyond understanding the evolutionary origins of resistance to encompass translational applications. For example, Paun et al. (2026) identified previously unknown genes in *Psychrobacter* SC65A.3 bacteria preserved in 5000-year-old cave ice, some of which encode molecules with inhibitory activity against modern multidrug-resistant pathogens, including MRSA and *E. coli* [66]. This finding suggests that ancient microbial communities represent an untapped reservoir for novel antimicrobial strategies, and that the primary value of ancient resistome research may encompass both ecological understanding and the practical expansion of the antimicrobial armamentarium.

Deep-sea sediments and archaeological soils provide additional evidence of pre-anthropogenic resistance. A recent study demonstrated that deep-sea sediments dated 8008–23,931 years have yielded five antibiotic-resistant pathogenic bacteria (*S. epidermidis*, *S. warneri*, *A. viridans*, *B. cereus*, *S. haemolyticus*) carrying multiple ARGs with resistance comparable to current pathogens [67]. Moreover, archaeological soil from İnönü Cave, Turkey, spanning the Chalcolithic Age to Early Iron Age, contained tetracycline resistance gene *tetA*, class 1 integron *intl1*, and oxacillinase gene *blaOXA58* [68]. Holocene sedimentary chronosequences spanning 12,000 years also revealed ARG abundance and diversity peaking in the deepest, oldest layers, driven by natural microbial succession rather than anthropogenic influence [69].

### 4.3. Functional Conservation of Resistance Mechanisms Across Evolutionary Time

Resistance mechanisms identified include all four major classes: efflux pumps (most common across environments), enzymatic degradation (β-lactamases, macrolide kinases, glycosylases), target modification, and reduced permeability [57,58,59,70]. Bacitracin resistance was predominant in polar glacier forelands, followed by rifamycin, fosfomycin, vancomycin, tetracycline, and β-lactam resistance [71]. Deep permafrost dated to 5821 BP contained ARGs with diverse resistance mechanisms, though less abundant and diverse than anthropogenically impacted sediments [72].

These observations indicate that the ancient resistome was not a static repository of dormant genetic traits but rather a dynamic, interconnected system shaped by long-term microbial interactions, environmental pressures, and evolutionary exchange. Indeed, the persistence of homologous resistance mechanisms across disparate deep-time environments and modern clinical pathogens implies sustained genetic mobility rather than independent evolutionary convergence. This continuity raises a critical question: how were the determinants of resistance maintained, diversified, and disseminated across microbial communities over geological timescales in the absence of anthropogenic antibiotic selection? Increasing evidence points to mobile genetic elements (MGEs) as the principal evolutionary machinery enabling the horizontal transfer, recombination, and long-term stabilization of ARGs within environmental microbiomes [73].

### 4.4. Ancient Origins and Environmental Reservoirs

The discovery of ARG-MGE correlations in the Arctic permafrost region showed that AMR is an ancient, ecologically-driven phenomenon rather than solely a consequence of modern antibiotic use [74]. Permafrost studies demonstrated that diverse resistance mechanisms existed at least 5000 years ago, with functional resistance genes predating anthropogenic antibiotic use [59]. The identification of eight MGE-associated ARGs in Arctic permafrost (predominantly phage-mediated) indicates that horizontal gene transfer has been maintaining and disseminating resistance determinants across geological timescales [74]. Significantly, deeper and older sediments show enhanced horizontal gene transfer potential, reflecting MGEs’ pivotal role in ARG evolution over millions of years [60,74]. This ancient resistome provides crucial baseline data for tracking the spread of modern resistance. The correlation between ARGs and MGEs (particularly *tnpA* transposases in glacier forelands) reveals the genetic machinery enabling resistance mobility [71]. Understanding that resistance genes in pristine environments differ from those in anthropogenically-impacted sites (with Arctic soils showing 100-fold lower ARG abundance than contaminated samples) allows differentiation between natural background resistance and human-driven selection [75,76].

These findings reveal that AMR originated as a natural defense mechanism in inter-microbial conflicts, with antibiotics serving as weapons against environmental bacteria long before their clinical use [60]. The environmental resistome serves as an abundant reservoir of potentially transferable resistance for pathogens, with soil metagenomes harboring the most diverse ARG pools [70]. Understanding this ancient origin is crucial for predicting the evolution of resistance and developing sustainable management strategies [58,77].

Therefore, from a deep-time perspective, the critical drivers of resistance emergence are not antibiotics per se, but selection pressure, transmission intensity, and ecological mixing. Antibiotics accelerated resistance by selecting from pre-existing genetic diversity and facilitating horizontal gene transfer, particularly under conditions of crowding, poor sanitation, and disrupted healthcare. These same conditions (documented repeatedly in the archaeological record) have historically accompanied periods of ecological stress, migration, urbanization, and conflict. Thus, AMR can be understood as an evolutionary response that becomes pathogenic when biosocial configurations favor amplification and spread.

This reframing aligns closely with syndemic theory. Resistance does not emerge in isolation but clusters with other vulnerabilities, including malnutrition, chronic infection, immune compromise, and breakdown of water, sanitation, and hygiene (WASH) infrastructure [9]. Conflict-affected and displaced populations exemplify this dynamic via healthcare disruption, unregulated antimicrobial use, incomplete treatment courses, and environmental contamination, which converge to create ideal conditions for resistance amplification [78,79,80]. These syndemic configurations parallel historical contexts in which infrastructure collapse and population aggregation likely facilitated the persistence and diversification of resistant strains long before the antibiotic era.

Importantly, deep-time resistome research highlights the central role of the environment as both a source and conduit for resistance genes. Soil, water, and animal-associated microbiomes function as interconnected reservoirs through which resistance circulates via MGEs. Modern surveillance systems, however, remain overwhelmingly focused on clinical isolates, neglecting environmental resistomes, particularly in low-resource, conflict-affected, and ecologically degraded settings [80,81,82].

### 4.5. Strategies to Fight AMR and Multidrug Resistant (MDR) Strains

With the increasing trend of antimicrobial resistance in various bacterial infectious diseases, several research teams worldwide are developing new strategies to combat multidrug-resistant (MDR) strains. These strategies include targeting new bacterial ultrastructures and metabolisms, using bacteriophages, and employing intelligent techniques to deliver antibiotics into bacterial cells.

#### 4.5.1. Targeting Bacterial Ultrastructures

Several structures in bacterial cells are well-known targets of various antibiotics; these include the bacterial cell wall, ribosomes, and cytoplasmic membrane. New ultrastructures are known to increase bacterial virulence, such as bacterial metallophores, these small molecules that chelate and import metals for bacteria under minimal conditions, such as in bacterial hosts. These metallophores play an important role in increasing bacterial virulence in both Gram-positive and Gram-negative bacteria [83,84,85,86,87]. That is why this ultrastructure and its metabolism are considered among the potential new targets for antimicrobial therapy [88].

#### 4.5.2. The Use of Bacteriophages

A new term has emerged in microbiology, mainly for targeting bacterial resistance to antibiotics: “Antimicrobial Bacteriophages.” Bacteriophages, or simply phages, are a specific family of viruses that can infect and kill bacteria. Several research teams are now working on different bacteriophages to treat bacterial infections by MDR strains [89].

#### 4.5.3. Trojan Horse Technique

This technique is well known as an efficient strategy that permits the introduction of an antibiotic inside a given bacterial cell by deceiving it [90,91].

### 4.6. Implications for Predicting Resistance Evolution

Integrating ancient resistome insights into One Health frameworks has direct implications for modern AMR control. If resistance is fundamentally ecological, then effective mitigation requires reducing selective pressure, limiting ecological mixing, and interrupting transmission pathways across human, animal, and environmental systems. Deep-time evidence reinforces calls for upstream, preventive strategies (including improved sanitation, environmental stewardship, antimicrobial governance, and conflict-sensitive health system strengthening) rather than reliance on pharmaceutical innovation alone. Recent evidence from ongoing conflicts demonstrates that strain on healthcare systems and disrupted infection prevention, diagnostics, and surveillance have coincided with increased detection of multidrug-resistant organisms, emphasizing that AMR dynamics in war-affected regions extend beyond traditional clinical surveillance [92]. Importantly, comparisons of conflict-affected countries with non-conflict settings reveal significantly fewer AMR surveillance sites and reporting to global monitoring frameworks, indicating systemic neglect of broader environmental and community resistomes in these contexts [80,81,93].

The deep-time resistome evidence demonstrates that AMR is fundamentally ecological and becomes clinically dangerous under specific biosocial conditions: crowding, poor sanitation, disrupted healthcare, and ecological mixing. These conditions are precisely those created by armed conflict and forced displacement, which function as powerful contemporary syndemic drivers, as examined in the following section.

## 5. Conflict, Displacement, and Sanitation Collapse as Recurring Syndemic Drivers of Infectious Disease

Armed conflicts act as powerful syndemic drivers of infectious disease emergence by simultaneously disrupting multiple interconnected systems: healthcare infrastructure, WASH services, nutrition, vaccination programs, and population stability. This creates conditions where disease clustering and adverse biological interactions markedly amplify morbidity and mortality [94,95,96].

### 5.1. Syndemic Mechanisms in Conflict Settings

Syndemic theory describes that conflict-associated disease outbreaks result not from single pathogens but from multi-pathogen burdens interacting with chronic disease, nutritional deficiency, and psychological trauma [9,96]. In fact, war creates environments of “syndemic vulnerability” where upstream social, economic, and structural determinants converge to produce concurrent and deleteriously interacting health adversities. These interactions generate feedback loops where disease burden reinforces social fragility, perpetuating conditions for further outbreaks.

### 5.2. WASH Infrastructure Collapse as Central Node

The destruction of WASH systems represents a critical mechanistic node within conflict syndemics [94,95,97]. The bombing of water facilities in Yemen, for example, enabled a cholera outbreak affecting over 500,000 people, predominantly children [94]. In Tigray, Ethiopia, war reduced improved water source access to 67.7% and sanitation coverage to 43.9%, with childhood diarrhea prevalence reaching 25.5%, directly correlated with unimproved water sources and latrine types [98]. As a consequence, WASH collapse facilitates fecal–oral transmission, environmental contamination, and sustained circulation of enteric pathogens, including cholera, typhoid, and hepatitis E.

### 5.3. Malnutrition–Infection Synergism and Antimicrobial Resistance Amplification

Conflict zones serve as hotspots for the emergence and dissemination of MDR organisms [97,99]. A systematic review found high colonization and infection rates with MDR bacteria in 21st-century conflicts, with Eastern Ukraine reporting particularly high rates of New Delhi metallo-β-lactamase producers [99]. In Western Ukraine’s evacuation pathway, Lviv exhibited significantly higher carbapenem resistance rates than Kyiv across multiple pathogens (MRSA, carbapenem-resistant *Klebsiella pneumoniae*, *E. coli*, *Pseudomonas aeruginosa*, *Acinetobacter* species), indicating potential for cross-border AMR spread [100]. Syrian conflict data also reveal high MDR rates among war-wounded patients, with 44.6% MDR Enterobacterales, 44.6% MRSA, and multivariable analysis showing higher MDR odds in Iraqi versus Syrian patients [80,82,101]. The rationale from those examples is that fragmentation of the healthcare system limits diagnostics, antimicrobial stewardship, and infection prevention, thereby promoting both uncontrolled transmission and resistance amplification.

Notably, the same structural determinants that render conflict zones hotspots for MDR infections (health system collapse, overcrowding, interrupted supply chains, and population displacement) also drive acute food insecurity and famine. Together, AMR and starvation represent interlocking manifestations of conflict-induced syndemic vulnerability rather than discrete humanitarian crises [102].

The Middle East offers another example of conflict-induced syndemic vulnerability, in which protracted political instability, episodic armed conflict, economic collapse, and mass displacement have eroded health care delivery, disrupted medication supply chains, and undermined surveillance and infection-prevention systems [103,104,105]. Restoring stability in all such conflicts is essential to prevent further deterioration in population health and to reduce conditions that favor the clustering of infectious diseases and the emergence and spread of MDR organisms in fragile health systems [105]. Importantly, the WHO has warned that conflict-driven healthcare system failures create an ideal ecological niche for MDR pathogen transmission, persistence, and cross-border spread, positioning AMR as a downstream biological consequence of famine and war rather than a parallel crisis [106].

### 5.4. Deep-Time Perspective and Preparedness Implications

Taken together, conflict-driven malnutrition and the collapse of food systems act synergistically with disrupted WASH services, healthcare infrastructure, and population displacement to amplify infectious disease risk through tightly coupled biological and social pathways. Immune suppression induced by undernutrition not only increases susceptibility to individual pathogens but also facilitates co-infection, prolonged carriage, and heightened transmission in overcrowded, environmentally contaminated settings. These processes exemplify syndemic amplification, whereby nutritional deprivation, infrastructural breakdown, and infectious disease reinforce one another in self-perpetuating feedback loops.

Reframing conflict-associated outbreaks through a syndemic lens shifts emphasis from pathogen-specific emergency responses to prevention strategies that target recurrent structural drivers: WASH infrastructure protection, continuity of primary healthcare, antimicrobial governance, and displacement mitigation [95,96]. The 19.7% of cholera outbreaks in Nigeria and 12.3% in the Democratic Republic of Congo attributable to conflict underscore the value of rapid assistance during conflict-associated outbreaks and addressing preexisting vulnerabilities like poverty and healthcare access [107].

Conflict-driven syndemics thus exemplify how structural vulnerability, rather than any single pathogen, determines disease outcomes in contemporary crises, mirroring patterns documented throughout the archaeological record. Climate change amplifies these dynamics by functioning as a force multiplier across all syndemic drivers, simultaneously expanding vector ranges, disrupting water and sanitation systems, driving forced migration, and intensifying ecological disruption.

## 6. Climate Change as a Force Multiplier Across Time

Climate change functions as a force multiplier, amplifying existing ecological, biological, and social vulnerabilities rather than acting as an isolated causal factor, reshaping infectious disease landscapes through complex, nonlinear, and context-dependent mechanisms [108,109,110].

### 6.1. Vector-Borne Disease Expansion

Rising temperatures expand the geographic ranges of vectors, lengthen transmission seasons, and alter pathogen–vector–host dynamics through multiple pathways [108,109]. From 1950 to 2018, global climate suitability for dengue transmission increased by 8.9% for *Aedes aegypti* (*A. aegypti*) and 15.0% for *Aedes albopictus* (*A. albopictus*). In highland areas, malaria transmission suitability rose by 38.7% in the African region and 149.7% in the Western Pacific region [3,5,110]. In addition, the reproductive number (R0) for arboviral diseases increased 13% for *A. aegypti* and 7% for A. albopictus transmission compared to the 1950s baselines [5].

These effects are not purely climatic phenomena. Vector-borne disease emergence has historically coincided with land-use change, deforestation, irrigation, and settlement reorganization, processes that interact synergistically with climate variability [108,109]. In the Ethiopian highlands, for example, a temperature increase of 0.2 °C per decade exposed growing populations of immunologically naïve persons to malaria risk [109]. In addition, the 2015 Zika emergence in Brazil occurred during a severe drought and unusually high temperatures associated with El Niño, combined with short- and long-term warming trends [109].

### 6.2. Waterborne Disease Amplification Through Infrastructure Collapse

Extreme precipitation and flooding mobilize fecal pathogens from upland pastures, overwhelm combined sewers, causing overflow, and compromise WASH infrastructure [111]. Heavy rainfall flushes Cryptosporidium parasites from watersheds into surface water sources, where oocysts survive chlorination and disperse through distribution systems. Climate change magnifies inequities, with approximately 2 billion people lacking safely managed drinking water services, mainly in the Global South [111].

Rapid urbanization in low- and middle-income countries has expanded informal settlements with inadequate sewage and drainage systems, exposing residents to parasitic infections, bacterial diarrheal diseases, and leptospirosis during extreme rainfall events. Following Cyclone Idai in Mozambique (2019), floodwaters precipitated a threefold surge in cholera cases, predominantly affecting children under five [112]. In Malawi, Cyclone Ana’s flooding (2022) contaminated water sources, causing a cholera outbreak claiming over 1200 lives [112]. Positive associations have been found between ambient temperature and increased diarrheal disease following heavy rainfall and flooding [113].

### 6.3. Zoonotic Spillover Risk

Climate change increases the risk of cross-species viral transmission through species range shifts and novel ecological assemblages [114]. Modeling predicts that by 2070, species will aggregate in new combinations at high elevations, in biodiversity hotspots, and in areas of high human population density in Asia and Africa, causing cross-species transmission of viruses an estimated 4000 times [114]. Bats account for most of the novel viral sharing due to their unique dispersal ability and are likely to share viruses along evolutionary pathways, facilitating future human emergence [114].

Climate sensitivity is widespread across zoonotic diseases, with evidence of significant relationships in 69.1% of temperature effects, 63.5% of precipitation effects, and 53.6% of humidity effects [115]. In a study conducted by a team of researchers in the United Kingdom, positive effects of temperature and rainfall on disease risk were shown to be more common than negative effects (46.5% vs. 22.6% and 37.8% vs. 25.7%, respectively) [115], with the most consistent relationship being between temperature and vector-borne zoonoses [115].

### 6.4. Biodiversity Loss as an Amplification Mechanism

Biodiversity loss is associated with increases in disease-related endpoints, alongside chemical pollution, climate change, and introduced species [2]. Taxa most likely to be zoonotic hosts often proliferate in human-dominated landscapes, increasing spillover likelihood, whereas in less-disturbed areas these reservoir hosts are less abundant and non-reservoirs predominate [116]. Thus, biodiversity loss appears to increase the risk of human exposure to both new and established zoonotic pathogens.

A global risk analysis found that higher temperatures, higher annual precipitation, water deficits, land-use changes, human encroachment on forested areas, increased population and livestock density, and biodiversity loss all contribute to epidemic risk, with biodiversity loss showing a complex and nonlinear relationship [117].

### 6.5. Syndemic Interactions and Unequal Vulnerability

Climate change health impacts are disproportionately concentrated in regions affected by poverty, political instability, and weak governance, where adaptive capacity is limited [110,111,112]. In these settings, climate stress compounds conflict, displacement, malnutrition, and sanitation collapse, generating tightly coupled syndemic configurations [9,96].

The number of months suitable for malaria transmission increased by 39% between 1950-59 and 2010-19 in highland areas of the low human development index (HDI) group, threatening highly disadvantaged populations previously safer from this disease [5]. Add to that, climate change impacts on crop yield potential (6.0% reduction for maize, 3.0% for winter wheat, 5.4% for soybean, 1.8% for rice in 2020 relative to 1981–2010) expose rising food insecurity risk [5].

### 6.6. Climate-Driven Migration and Disease Transmission

Climate change is the sole contributing factor for at least 4400 people already forced to migrate worldwide, with estimates ranging from 25 million to 1 billion climate migrants by 2050 [110]. Climate-induced migration occurs through sea-level rise, coastal erosion, and changes in precipitation and temperature, reducing land arability and exacerbating food and water insecurity. Additionally, migration driven by climate change has severe impacts on mental and physical health, both directly and by disrupting essential health and social services [110].

### 6.7. Deep-Time Perspective and Prevention Strategies

The novelty of the current moment lies in the speed, scale, and global interconnectedness of climate-driven changes, which compress evolutionary, ecological, and social processes into shorter timeframes [118,119]. Changes in infectious disease prevalence reflect not only temperature, humidity, and weather-related phenomena on pathogens, vectors, and hosts, but also complex social and environmental factors, including land use, migration, and vector control [118].

Prevention strategies must prioritize ecological stewardship, social resilience, and infrastructure protection alongside biomedical innovation [111]. This includes protecting WASH infrastructure, ensuring primary healthcare continuity, implementing antimicrobial governance, and mitigating forced displacement [95,120]. Strengthening disease surveillance systems to incorporate climate data could enhance early warning capabilities, while national adaptation plans must prioritize health resilience by bridging gaps between water, agriculture, and infrastructure policies.

## 7. A Deep-Time Syndemic Framework for Pandemic Preparedness

A deep-time syndemic framework reconceptualizes pandemics as biological expressions of reproducible biosocial “outbreak modules” that have operated across millennia, positioning pathogens not as primary drivers but as responders to conditions created by human–environment interactions at specific ecological and social thresholds [9,96,121].

It is important to distinguish the deep-time syndemic framework from existing integrative models. While the One Health framework emphasizes the interconnectedness of human, animal, and environmental health at the contemporary interface, and ecosocial models focus on how social structures shape disease distribution in present populations, the deep-time syndemic framework uniquely incorporates empirical evidence spanning millennia to demonstrate that the biosocial configurations driving disease emergence today have deep evolutionary precedents. The framework does not claim direct predictive capacity for specific future outbreaks; rather, it identifies structurally analogous conditions (“outbreak modules”) that have repeatedly facilitated disease emergence across vastly different time periods and geographic contexts. This structural pattern recognition complements, rather than replaces, contemporary surveillance and modeling approaches by providing an evolutionary baseline against which modern risk configurations can be assessed. It is equally important to acknowledge that contemporary outbreak dynamics are shaped by variables, including global mobility, industrialized agriculture, and geopolitical instability, that may lack direct analogs in the archaeological record.

### 7.1. COVID-19 as a Syndemic

COVID-19’s global impact was shaped less by viral biology alone than by syndemic interactions involving chronic disease burden, socioeconomic inequality, labor precarity, housing density, and unequal healthcare access [110,118,119]. Mortality clustered disproportionately among marginalized populations (racialized communities, migrants, individuals with disabilities, and those with cardiometabolic comorbidities), consistent with syndemic amplification rather than uniform exposure [110,118].

In the United States, age-adjusted COVID-19 mortality rates were 2.0 times higher for Black persons, 2.3 times higher for Hispanic persons, and 2.2 times higher for American Indian/Alaska Native persons compared to White persons [122,123]. These disparities reflect structural determinants, including occupational exposure (essential workers unable to work remotely), housing density (multigenerational households), barriers to healthcare access, and a higher prevalence of comorbidities shaped by social determinants.

In addition, evidence from large cohorts and pooled analyses shows that host immunogenetic markers modestly shape COVID-19 risk within the broader syndemic context. Across multiple studies and meta-analyses, individuals with blood groups A, B, and AB and Rh-positive status experienced higher odds of SARS-CoV-2 infection than those with group O and Rh-negative status, and blood group A also showed a consistent association with more severe disease and higher mortality [124,125,126].

### 7.2. Chronic Disease Comorbidity Interactions

COVID-19 intersected with pre-existing noncommunicable disease epidemics, creating bidirectional syndemic effects. Diabetes conferred an increased risk of severe COVID-19, while COVID-19 infection increased new diabetes diagnoses [127]. Cardiovascular disease showed similar bidirectional relationships, with pre-existing cardiac conditions increasing COVID-19 severity and COVID-19 infection increasing subsequent cardiovascular events [128]. Obesity (BMI ≥ 30) was also associated with an increased risk of hospitalization, ICU admission, and mortality risk from COVID-19 [129,130]. In fact, the syndemic interaction between obesity and COVID-19 reflects shared pathophysiological mechanisms, including chronic inflammation, immune dysregulation, and metabolic dysfunction [131,132].

### 7.3. Mental Health Syndemic Interactions

COVID-19 precipitated a global mental health crisis through multiple pathways: direct neuropsychiatric effects of infection, psychological trauma from illness/bereavement, social isolation from public health measures, economic insecurity, and healthcare disruption [133,134,135]. Mental health conditions showed bidirectional syndemic relationships with COVID-19. Pre-existing psychiatric disorders were associated with increased COVID-19 infection risk and mortality [136,137]. Conversely, COVID-19 survivors showed an increased incidence of new psychiatric diagnoses, such as anxiety disorders and depressive disorders [136].

### 7.4. Healthcare System Cascade Failures

Health system overload disrupts routine care, vaccination programs, and antimicrobial stewardship, creating secondary waves of preventable morbidity. During 2020, routine childhood vaccination coverage declined globally, with an estimated 23 million children missing basic vaccines, the highest number since 2009, and 3.7 million more than in 2019 [138,139]. This disruption precipitated measles outbreaks in multiple countries. Cancer screening and diagnosis also declined substantially, with reductions of more than 90% for breast, colorectal, and prostate cancer screenings during lockdown periods [140,141]. Modeling suggests these delays will result in increased cancer mortality for years, with estimates of 3291–3621 excess breast cancer deaths over 5 years in England alone [141,142].

Moreover, antimicrobial governance programs were disrupted during the COVID-19 pandemic [143,144]. Antibiotic misuse was widespread and well-documented, with approximately 75% of hospitalized COVID-19 patients receiving antibiotics despite bacterial co-infection rates of only 3.5–8.6% [143,145,146,147,148,149]. The excessive and/or misuse of antibiotics, especially broad-spectrum antibiotics, during the COVID-19 pandemic has increased the rate of AMR among isolates in both community- and hospital-acquired infections [148,149]. This represents a substantial disconnect between prescribing practices and actual clinical need.

The prevalence of inappropriate antibiotic prescribing varied by region and time period [150]. In a Spanish registry of nearly 14,000 patients, 34.2% received inappropriately prescribed antibiotics [151]. Low- and middle-income countries (LMICs) showed particularly high rates, with antibiotic prescribing reaching 71.7% in China and 86.5% in other LMICs [152]. Global pharmaceutical sales data from 71 countries demonstrated that antibiotic sales were positively associated with COVID-19 case counts, with a 10% increase in monthly cases correlating with 0.3% higher combined antibiotic sales [150].

Several factors drove inappropriate prescribing. Early in the pandemic, diagnostic uncertainty due to limited SARS-CoV-2 testing capacity and delayed turnaround times led physicians to empirically treat for bacterial pneumonia [153]. Clinical presentations suggesting bacterial co-infection (including fever, dyspnea, elevated C-reactive protein, and flu-like symptoms) were independently associated with inappropriate prescribing [151]. Behavioral factors also played a role, as physicians’ desire to intervene for severely ill patients often superseded evidence-based practice, particularly given the abstract nature of AMR consequences compared to immediate patient concerns [151]. The consequences of this overuse included increased adverse drug reactions (4.9% vs. 2.7% in patients not receiving antibiotics) and concerns about accelerating AMR globally [151,153].

### 7.5. Operational Implications for Pandemic Preparedness

The deep-time syndemic framework reframes pandemic prevention as a challenge of structural resilience rather than one of reactive pathogen control alone [9,19]. From this perspective, effective preparedness requires addressing upstream social and economic determinants, such as housing security, living wages, universal access to healthcare, and occupational protections, that shape baseline syndemic vulnerability long before an outbreak occurs [154]. It also necessitates sustained protection of essential infrastructure, including WASH systems, primary healthcare, food security, and mental health services, which function as critical buffers against syndemic amplification during periods of crisis [135]. Equity-centered surveillance systems capable of real-time, disaggregated data collection are essential to reveal disproportionate burdens and guide targeted interventions, while integrated response frameworks must explicitly account for interactions between emerging infections, chronic disease management, mental health care, and antimicrobial governance [155]. Within this model, One Health implementation is not optional but foundational, recognizing that zoonotic spillover risk, AMR, and pandemic preparedness are inseparable from environmental integrity, animal health, and social determinants [6,12,16,17]. Importantly, the framework positions paleopathology and ancient genomics as forward-looking tools for pandemic anticipation, enabling identification of recurrent biosocial configurations that have repeatedly generated large-scale disease emergence across millennia and allowing predictive pattern recognition that transcends individual pathogen characteristics.

Translating deep-time evidence into operational public health practice requires distinguishing between recommendations that are immediately actionable and those that remain conceptual or long-term. Among immediately actionable strategies, integrating environmental resistome monitoring into existing AMR surveillance platforms represents a feasible extension of current wastewater and environmental metagenomics programs; ancient resistome baselines can help calibrate what constitutes background versus anthropogenically amplified resistance in environmental samples. Similarly, incorporating archaeological and paleogenomic data on historically recurring outbreak modules into risk-mapping tools could enhance existing geographic information system-based early warning frameworks by flagging regions where multiple syndemic drivers converge, such as areas undergoing rapid land-use change and adjacent to high-density livestock production and weakened health infrastructure. Surveillance system design can also benefit from deep-time insights by prioritizing monitoring at human–animal–environment interfaces that have historically generated spillovers, particularly during periods of ecological stress or population displacement. In contrast, longer-term and more conceptual applications include developing predictive models that integrate paleogenomic outbreak module data with contemporary climate projections and land-use change scenarios to anticipate future zones of heightened spillover risk, as well as embedding evolutionary baselines into One Health governance frameworks to establish thresholds for preemptive intervention. Large-scale archaeogenomic screening studies, such as those demonstrating temporal clustering of zoonotic pathogen emergence following domestication, provide proof-of-concept that deep-time data can inform the temporal and spatial calibration of modern surveillance, though the infrastructure for routine integration of such data into public health decision-making remains to be developed.

## 8. Knowledge Gaps and Future Directions

Despite rapid advances in paleopathology, ancient pathogen genomics, and the One Health conceptual framework, critical gaps remain in translating deep-time insights into actionable frameworks for contemporary infectious disease prevention. Current ancient pathogen datasets are geographically skewed toward Eurasia, with persistent underrepresentation of Africa, the Americas, Oceania, and much of South and Southeast Asia, regions that now shoulder a disproportionate burden of emerging infections [24,156]. This imbalance constrains inference regarding pathogen evolution, zoonotic emergence, and syndemic dynamics in precisely the settings where such knowledge is most urgently needed [13,36].

Temporal resolution represents an additional limitation. Many datasets insufficiently capture periods of rapid ecological and sociopolitical change, such as climatic instability, urban collapse, imperial expansion, or intensified conflict, when disease emergence may have been driven by abrupt structural disruption rather than gradual ecological drift. High-resolution sampling across such transitions is essential to distinguish background pathogen evolution from threshold effects with direct relevance to modern early-warning systems [24].

Equally important is the need to situate ancient genomic findings more consistently within their broader biosocial contexts. Syndemic interpretation requires systematic integration of archaeological indicators of mobility, settlement density, subsistence strategies, sanitation infrastructure, warfare, and inequality (data that are often available but underutilized). Without this integration, pathogen reconstructions risk biological reductionism and lose explanatory power for contemporary disease emergence [19].

Future research should therefore prioritize interdisciplinary designs that explicitly link pathogen diversity, co-infection, and resistance profiles with indicators of social stress and environmental disruption. Both ancient and modern outbreaks are increasingly recognized as multi-pathogen phenomena shaped by immune modulation, nutritional status, and ecological exposure rather than single agents acting in isolation. Scaling multi-pathogen screening and integrating ancient host genomic data (particularly immune-related loci) will be critical for reconstructing how susceptibility and disease burden evolved under different biosocial configurations [9,96].

A major translational gap also lies in connecting deep-time findings to modern surveillance and preparedness systems. Although wastewater monitoring, environmental metagenomics, and wildlife surveillance expanded rapidly after COVID-19, these efforts rarely incorporate evolutionary baselines derived from paleopathology. Integrating ancient pathogen and resistome data into contemporary surveillance could improve risk stratification, calibrate thresholds, and help distinguish structural shifts in disease ecology from background variability [15]. Add to that, emerging strategies to combat AMR include targeting bacterial metallophores (metal-chelating molecules essential for bacterial iron acquisition and virulence) and employing “Trojan Horse” antibiotic delivery systems that exploit bacterial nutrient uptake pathways [90,157]. Metallophores, particularly siderophores, are small molecules that bacteria produce to sequester iron from the environment, a process critical for growth and pathogenicity [158]. Targeting siderophore biosynthesis or hijacking siderophore transport systems represents a promising dual approach: inhibiting siderophore production can starve bacteria of essential iron, while siderophore-antibiotic conjugates can actively transport antimicrobial agents across otherwise impermeable bacterial membranes [159].

The Trojan Horse strategy conjugates antibiotics to siderophores, antimicrobial peptides, or monoclonal antibodies, enabling active transport into bacterial cells and circumventing permeability barriers that limit conventional antibiotic efficacy, particularly against Gram-negative pathogens [90,157,160]. Cefiderocol, a cephalosporin-siderophore conjugate, exemplifies this approach’s clinical potential, demonstrating potent activity against carbapenem-resistant and multidrug-resistant Gram-negative bacteria, and has received FDA approval [158,159,161]. Siderophore–antibiotic conjugates can also repurpose older antibiotics with limited clinical utility due to poor penetration or toxicity, potentially expanding the therapeutic arsenal against resistant pathogens [90,162]. Beyond siderophores, conjugation with other bacterial uptake substrates (including vitamin B12, carbohydrates, and amino acids) offers additional pathways for targeted antibiotic delivery [163]. These strategies represent a substantial shift from developing entirely new antimicrobial scaffolds to exploiting bacterial physiology to enhance drug delivery, potentially reducing the development of resistance while reinvigorating existing antibiotics [124,164].

## 9. Conclusions

This review argues that emerging and re-emerging infectious diseases are best understood not as isolated biological events but as predictable outcomes of recurring biosocial configurations that have structured human–pathogen interactions across time. From the Late Pleistocene through the Holocene, disease risk repeatedly intensified when ecological disruption, population mobility or displacement, sanitation collapse, and governance failure converged. Those conditions are now re-emerging under climate change, armed conflict, rapid urbanization, and widening social inequities [9,13,19].

Ancient DNA and paleomicrobiological evidence further demonstrate that AMR predates modern antibiotic use, reframing AMR as an ancient ecological phenomenon amplified (rather than created) by contemporary selection pressures. This insight reflects the limits of pharmaceutical solutions alone and reinforces the need for preventive strategies addressing environmental stewardship, sanitation, and health-system resilience [77,165].

An important conceptual tension warrants explicit acknowledgment: evolutionary biology suggests that infectious diseases, including large-scale outbreaks, are structurally recurrent outcomes of host–pathogen interactions under ecological pressures, population densities, and transmission opportunities. Pandemics can therefore be understood not only as preventable events but also as phenomena emerging from complex adaptive systems that may not be fully avoidable. The deep-time syndemic framework serves a dual purpose; it provides explanatory insight into these recurring processes while simultaneously identifying modifiable structural determinants (sanitation infrastructure, health system resilience, ecological stewardship, and social equity) that can reduce the frequency, severity, and inequitable distribution of outbreaks. We emphasize that the policy implications presented in this review represent logical extensions of the evidence base rather than empirically validated interventions; they warrant further investigation through targeted research and stakeholder engagement. Additionally, it must be acknowledged that contemporary conditions in the Anthropocene, including the unprecedented scale of genetically homogeneous livestock populations, the pace of ecological disruption, and global interconnectedness, represent qualitative departures from earlier epochs, potentially generating novel disease dynamics without direct historical precedent.

Ultimately, paleopathology and ancient pathogen genomics should be viewed not as retrospective disciplines but as essential components of a forward-looking global health strategy. A deep-time syndemic framework shifts pandemic preparedness away from reactive pathogen prediction toward identification and mitigation of structural risk configurations. By revealing which biosocial conditions have repeatedly generated large-scale disease emergence across history, deep-time evidence offers a realistic foundation for prevention, resilience, and coordinated global response in an era of accelerating planetary change.

## Data Availability

No new data were created or analyzed in this study. Data sharing is not applicable to this article.

## Abbreviations

The following abbreviations are used in this manuscript:

aDNA	ancient DNA
AI	Artificial intelligence
AMR	Antimicrobial resistance
ARG(s)	antimicrobial resistance gene(s)
BCE	Before common era
BMI	Body mass index
BP	Before present
COVID-19	Coronavirus disease 2019
DNA	Deoxyribonucleic acid
FAO	Food and Agriculture Organization
FDA	Food and Drug Administration
HDI	Human development index
ICU	Intensive care unit
LMICs	Low- and middle-income countries
MDR	Multidrug-resistant
MGE(s)	Mobile genetic elements(s)
MRSA	Methicillin-resistant *Staphylococcus aureus*
NGS	Next generation sequencing
OHHLEP	One Health High-Level Expert Panel
PCR	Polymerase chain reaction
R0	Basic reproductive number
SARS-CoV-2	Severe acute respiratory syndrome coronavirus 2
TB	Tuberculosis
UN	United Nations
UNEP	UN Environment Programme
WASH	water, sanitation, and hygiene
WHO	World Health Organization
WOAH	World Organization for Animal Health
